# Supplementary material for: Authentic self-expression on social media is associated with greater subjective well-being
Source: Nat Commun. 2020 Oct 6;11:4889. doi: 10.1038/s41467-020-18539-w (PMC7538578; doi:10.1038/s41467-020-18539-w)
Supplement: Supplementary file 1 — Supplementary Information [file 41467_2020_18539_MOESM1_ESM.pdf]

Authentic Self-Expression on Social Media is Associated with Greater Subjective Well-Being

(Bailey et al., 2020)

Contents:

Overview

A. Supplementary Methods – Study 1 (pg. 3)
B. Supplementary Methods – Study 2 (pg. 5)
C. Supplementary Notes – Study 1 (pg. 8)
D. Supplementary Notes – Study 2 (pg. 12)
E. Supplementary Figures (pg. 14)
F. Supplementary Tables – Study 1 (pg. 18)
G. Supplementary Tables – Study 2 (pg. 23)
H. Supplementary References (pg. 25)

List of Figures

Supplementary Figure 1 Study 1 Response Surface Plots of Interaction of QA and Personality Traits.
Supplementary Figure 2 Study 1 Response Surface Plots of the Interaction of QA and Personality Traits.
Supplementary Figure 3 Study 1 Standardized Ridgeline Plots of QA.
Supplementary Figure 4 Study 1 Relationship between Life Satisfaction and Deviations from Self-Reports.

List of Tables

Supplementary Table 1 Study 1 Correlation Table, Likes-Based Model.
Supplementary Table 2 Study 1 Correlation Table, Language-Based Model.

Supplementary Table 3 Study 1 Regression Analysis of Life Satisfaction on Quantified Authenticity.

Supplementary Table 4 Study 1 Regression Analysis of Life Satisfaction on Four-QA Measures.

Supplementary Table 5 Study 1 Regression Analysis of Life Satisfaction on Common Participants.

Supplementary Table 6 Study 1 Correlation Table of Common Participants

Supplementary Table 7 Study 2 Attrition.

Supplementary Table 8 Study 2 Key Outcome Variables By Condition.

Supplementary Table 9 Study 2 Week 1 Well-Being Outcomes By Condition.

A. Supplementary Methods – Study 1

Quantified Authenticity Measures. In Study 1, to test whether Quantified Authenticity predicted Life Satisfaction (H1) we regressed Life Satisfaction on the Likes-based and Language-based measures of QA using linear regression analyses (Model 1, Supplementary Table 4). In Model 2, we added personality extremeness as well as the main effects of self-reported personality to control for the fact that personality traits are known to be associated with Life Satisfaction[1–4]. Based on the recommendation of Simonsohn, Simmons & Nelson[5] we further tested the robustness of our effects by estimating Quantified Authenticity using three additional measures: Manhattan Distance, Cosine Similarity and Correlational Similarity. The standardized effects and standard errors of each were plotted in Figure 1 of the manuscript. Second, we analyzed whether participants with more socially desirable profiles benefit more from behaving authentically on Facebook with regards to their Life Satisfaction (H2). To this end, we added the interaction effects of Quantified Authenticity with each of the Big Five traits to the regression of Life Satisfaction (Model 3, Supplementary Table 4).

The robustness of our findings was tested by calculating various types of distance and similarity measures. We consider the robustness of Quantified Authenticity by calculating Manhattan distance, cosine similarity and correlational similarity as below.

The second distance measure we utilized is a Manhattan Distance measure. Manhattan Distance uses a grid-like measure of distance between two points. Distance is calculated as the sum of the horizontal and vertical distances between two points over the dimensions of the vectors, see Supplementary Equation 1.

$$Supplementary\ Equation\ 1$$

$$Manhattan\ Distance\ (x, y) = 0 - \sum_{i}^{n} |\ x_i - \ y_i\ |$$

Cosine Similarity was calculated for each metric of interest. This measure is based on the cosine angle between two vectors and captures overall orientation. Cosine Similarity can be thought of as capturing overall cohesion and is frequently used in language-based similarity modeling. See Supplementary Equation 2.

$$Supplementary\ Equation\ 2$$

$$Cosine\ Similarity\ (x, y) = \frac{\sum_{i}^{n} x_i y_i}{\sqrt{\sum_{i}^{n} x_i^2}\sqrt{\sum_{i}^{n} y_i^2}}$$

Finally, a measure of Correlational Similarity was calculated. Correlational Similarity measures angular separation of two vectors for centered x and y coordinates. See Supplementary Equation 3.

$$Supplementary\ Equation\ 3$$

$$Correlational\ Similarity\ (x, y) = \frac{\sum x_i y_i}{\sqrt{\sum_{i}^{n} x_i \sum_{i}^{n} y_i}}$$

B. Supplementary Methods – Study 2

In Study 2, A total of 200 participants were recruited to be part of the study. Overall, we observed an attrition rate of 35.50% over the two week study. As defined in our preregistration plan, we excluded participants who indicated at the end of the last study that they did not take the study seriously (N = 39; See Supplementary Table 7).

Treatment Text and Examples. All participants in Study 2 received both an authenticity and an idealized treatment. They were randomly assigned to receive either the authenticity or the idealized treatment first, followed by the opposite treatment. The exact treatment text for the Authenticity manipulation is presented below:

"When we sent you the link to this study, we also sent you a report that provides personalized feedback on your personality. This report was generated based on the responses you submitted in the pre-screening. If you haven't looked at it yet, please read it carefully right now. This feedback reflects how you see yourself. You can also consider it your "true" self.

For the next week, we would like you to ask you to post on social media in a way that presents a more authentic version of yourself. What would the real version of yourself look like that you'd be proud to present to your friends and other people reading your posts? Regardless of whether you usually post this way, please craft your messages for the next week such that they portray an authentic, real or true version of yourself, your feelings, and your experiences To help you achieve this goal, we would like to ask you to list three ways in which you could present a version of yourself on social media that is more authentic and true to yourself than what you usually post (e.g. you could try to be show others how outgoing and social you are, or how much you appreciate you appreciate your "me time"). You can refer back to the feedback report which describes how you really see yourself."

The exact text used for the idealized treatment is presented below:

"When we sent you the link to this study, we also sent you a report that provides personalized feedback on your personality. This report was generated based on the responses you

submitted in the pre-screening. If you haven't looked at it yet, please read it carefully right now. This feedback reflects how you see yourself. You can also consider it your "true" self.

For the next week, we would like you to ask you to post on social media in a way that presents a more idealized version of yourself. What would the ideal version of yourself look like in the eyes of your friends and other people reading your posts? Regardless of whether you usually post this way, please craft your messages for the next week such that they portray an enhanced, popular or perfect version of yourself. To help you achieve this goal, we would like to ask you to list three ways in which you could present a version of yourself on social media that is more idealized and socially desirable than what you usually post (e.g. you could try to be seen as more outgoing and social, or more organized). You can refer back to the feedback report which describes how you really see yourself."

Sample responses to the prompts are presented below. Responses to the authenticity treatment include:

"I would post photos of myself trying new things (i.e. eating interesting food, visiting a new place) to be authentic to my Openness, which is a trait I consider positive."

"While interacting with social media this week, instead of trying to focus on making everything perfect (choosing the right caption or the right image based on what I think people want to see) I should instead focus on expressing what I truly feel and sharing the moments that truly make me happy!"

"I'm also very Conscientiousness and agreeable, and that has made me think twice before calling people out or getting into arguments online/posting personal opinions that others might disagree with, but I should be able to post without thinking so deeply about these factors first."

Sample responses to the prompts are presented below. Responses to the idealized treatment include:

"Referring to the Conscientiousness section, I could play on my perfectionism and post organized/aesthetic photos of my study spaces. Such neat photos might appear perfect to others, even if my study spaces aren't actually normal neat and the photos are only staged."

"It looks like I could work on being more agreeable, and I think I read somewhere people gravitate towards pictures with smiling (e.g. agreeable) people. So I could post more pictures in which I am smiling."

"I got a high score on Neuroticism - to remain appealing on social media, I should keep negative posts to a minimum because people don't like seeing negative posts unless they are funny or relatable."

C. Supplementary Notes – Study 1

Supplementary Tables 1 and 2 present the means, standard deviations, and correlations between the measures of Quantified Authenticity based on Euclidean Distance, Manhattan Distance, Correlational Similarity, and Cosine Similarity from Study 1. In both comparison points, the QA measures are positively and significantly correlated with one another.

The effects of Quantified Authenticity on Life Satisfaction when controlling for age and gender. Supplementary Table 3 presents the results from Study 1 with additional control variables. For a subset of our full dataset, we were able to obtain age and gender controls. This included 6,648 participants in the Likes-based model (61.24% female, mean age = 24.97 years, SD age = 8.42 years) and 2,943 participants in the Language-based model (58.27% female, mean age = 25.97 years, SD age = 10.42 years). We found that in the Likes-based model the effect of QA predicting Life Satisfaction was robust to the inclusion of gender and age controls in both Models 1 and 2. In the Language-based model, the effect of QA on Life Satisfaction remained significant in Model 1 but became non-significant in Model 2.

Additionally, Supplementary Table 3 presents the interaction effects of QA on demographic controls as well as personality traits discussed in the main text. As with the results for personality traits, we find inconsistent or null effect of the interaction between age and Quantified Authenticity, and gender and Quantified Authenticity on Life Satisfaction. The only significant demographic interaction (age) was found in the Likes-based model but did not replicate in the Language-based model.

The effects of Quantified Authenticity for the overlapping sample of Likes and Language participants. The samples referenced in the Likes-based and Language-based models differed depending on the access privileges the users selected, and the amount of required Likes

and Status updates for the personality prediction models[6,7]. To provide additional evidence for the robustness of the effect, we limited the sample of users to only those who are included in both samples, resulting in a subset of 1,711 participants. In Supplementary Table 5, we replicate the results of QA (calculated as the inverse Euclidean Distance) on Life Satisfaction in this subset of participants.

The effects of Quantified Authenticity on the level of individual personality traits. To test whether authentic self-expression on certain personality traits might be more important for Life Satisfaction than others, we investigated the interactions between self-reported and predicted traits using response surface analyses. Supplementary Figures 1 and 2 visualize the interactions for the Likes-based and Language-based models respectively. While the findings generally support the authenticity hypothesis, they also highlight the fact that when looking at individual traits there might indeed be evidence for the proposition that people with socially desirable traits benefit from authentic self-expression. People scoring low on Conscientiousness, for example, do not seem to experience a boost in life satisfaction when having a Like-profile that is low on Conscientiousness, while people scoring high on Conscientiousness do see a boost in life satisfaction when their profile is aligned with their personality. The same pattern is observed for a number of different instances, indicating that being more authentic might not necessarily benefit individuals with less socially desirable profiles.

The Relationship of Quantified Authenticity between Samples. In order to evaluate the robustness of the Quantified Authenticity (QA) variable, we compared QA calculated based on Likes and Language in users that were common in both samples (N = 1,211) described above. In Supplementary Table 6, we show the correlations between QA in both samples, as well as the predicted personality traits from each model. We find that both QA measures (Likes-based and

Language-based) are positively and significantly correlated with one another. Additionally, all five of the predicted traits from the Likes-based model are positively and significantly correlated with the same trait from the Language-based model.

Differential effects of normative self-enhancement. As we outlined in the description of our measures in the main body of the manuscript, we conceptualize authenticity as a continuum from high authenticity (i.e. low discrepancy between self-reported and estimated personality profiles) to low authenticity/self-idealization (i.e. high discrepancy between self-reported and estimated personality profiles). Instead of normatively defining which directions of the personality traits an individual would be inauthentic towards (for instance, low Neuroticism). This conceptualization assumes that individuals have an inherent drive to self-enhance on social media, and therefore any deviation from the true self is a reflection of self-idealizing behavior. However, given the existing literature on socially desirable personality profiles – which show that, on average, high Openness, Extraversion, Conscientiousness and Agreeableness, but low Neuroticism are desirable.[8] As a robustness check, here we isolate the extent to which deviation from self-reported personality presents as idealization by separating idealization from self-depreciation on Life Satisfaction, and illustrating their effects on Life Satisfaction separately.

Specifically, we first calculated the difference between the self-reported and predicted personality traits such that positive and higher values indicate higher levels of self-idealization, and negative and lower values indicate higher levels of self-deprecation (i.e. subtracting the self-reported from the predicted score for Openness, Conscientiousness, Extraversion and Agreeableness, and subtracting the predicted score from the self-reported score for Neuroticism). A score of zero would indicate that there is no deviation in the self-reported and predicted personality-profiles of a participant.

As Supplementary Figures 4A (Likes-based model) and 4B (Language-based model) show, the effect of authentic self-expression on Life Satisfaction was particularly pronounced for the normative self-enhancing aspect of Quantified Authenticity. That is, higher levels of self-enhancement towards socially desirable personality traits were associated with lower levels of Life Satisfaction.

D. Supplementary Notes – Study 2

Positive and Negative Affect Additional Analyses. The pre-registration plan for Study 2 is available on OSF.[9] We note an error in the pre-registration plan which resulted from an oversight of one of the authors when setting up the online survey: the pre-registration incorrectly referred to wrong citation as the positive and negative affect measure. As mentioned in the manuscript, participants completed the 16-item Brief Mood Introspection Survey instead of the PANAS scale which was pre-registered. Past research has found that these two scales are highly correlated[10], predict similar outcomes[11–13], and can be combined with acceptable internal reliability[14]. Additionally, we returned to the PANAS-X handbook to review the similarities between the two scales. We found that there were seven items (four items for positive affect: lively, happy, content, active; three items for negative affect: sad, jittery, nervous) which were shared between the two scales. We extracted these items to create second measures positive affect and negative affect and test the replication of our effects on only these items in the Results section below.

Additionally, we considered how the 7-item PANAS measure performed against our hypotheses. At t0, the measure of positive affect as measured by the BMIS had good consistency (alpha = .81), as did the measure of negative affect (alpha = .82). The 4-item measure of PANAS positive affect also had internal reliability (alpha = .77). The 3-item measure of negative affect based on PANAS had lower internal reliability than the BMIS measure, but was still at an acceptable level (alpha = .64). Additionally, we found that the subset of scales was positively related to the overall positive affect scale reported in the manuscript (r = .93, p < .001) and the subset of negative affect was strongly and positively related to the overall negative affect scale reported in the manuscript (r = .86, p < .001).

Using the PANAS subset, we replicated our main experimental outcomes such that within participants differences were found. In the authentic week, participants reported significantly higher positive affect compared to their self-reported affect in the self-idealized week (mean difference = .28 [.098, .469], $t = 3.04$, $p = .003$). Additionally, we found marginally significantly lower effects for negative affect (mean difference = -.21 [-.448, .033], $t = -1.71$, $p = .091$). While looking at the responses in t2, we also found a significant difference between conditions such that the group which received the authenticity treatment had greater positive affect (mean difference = .58 [.96, .20], $t = 3.01$, $p = .004$). Lower negative affect was not significant (mean difference = -.30 [-.817, .218], $t = -1.15$, $p = .254$). This suggests that the BMIS measure of positive and negative affect performs similarly to the PANAS measures of the same affective outcomes.

Results of key outcome variables on condition assignment for week 1 are presented in Supplementary Table 8. Results of key outcome variables on condition assignment for week 2 are presented in Supplementary Table 9 with robustness checks for demographics (age and gender) and main effects of the Big Five personality traits.

E. Supplementary Figures

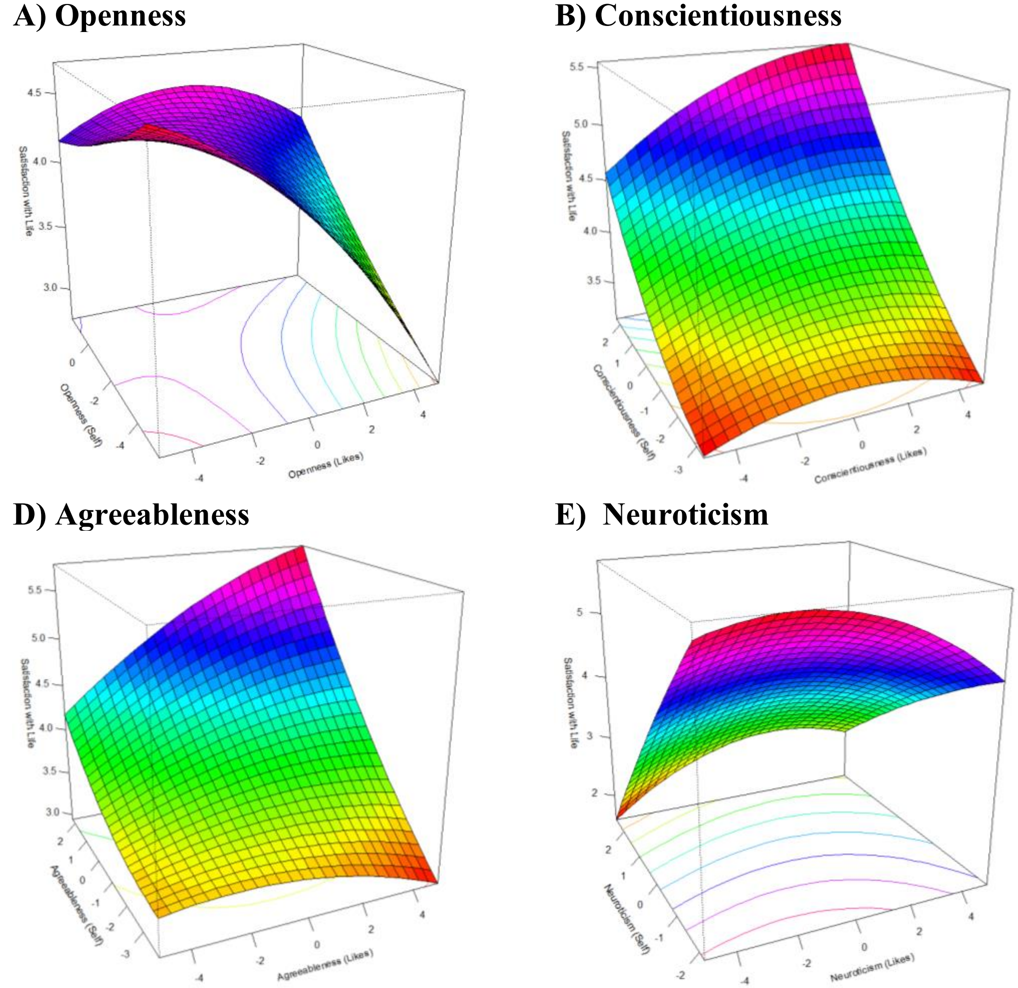

Supplementary Figure 1 Study 1Response Surface Plots of Interaction of QA and Personality Traits. Figures represent the response surface plots of the interactions between Quantified Authenticity and Self-reported and Likes-predicted Personality Traits on Life Satisfaction. Graphs from left-top to bottom-right present the interaction of QA and the following traits: Openness, Conscientiousness, Extraversion, Agreeableness, and Neuroticism.

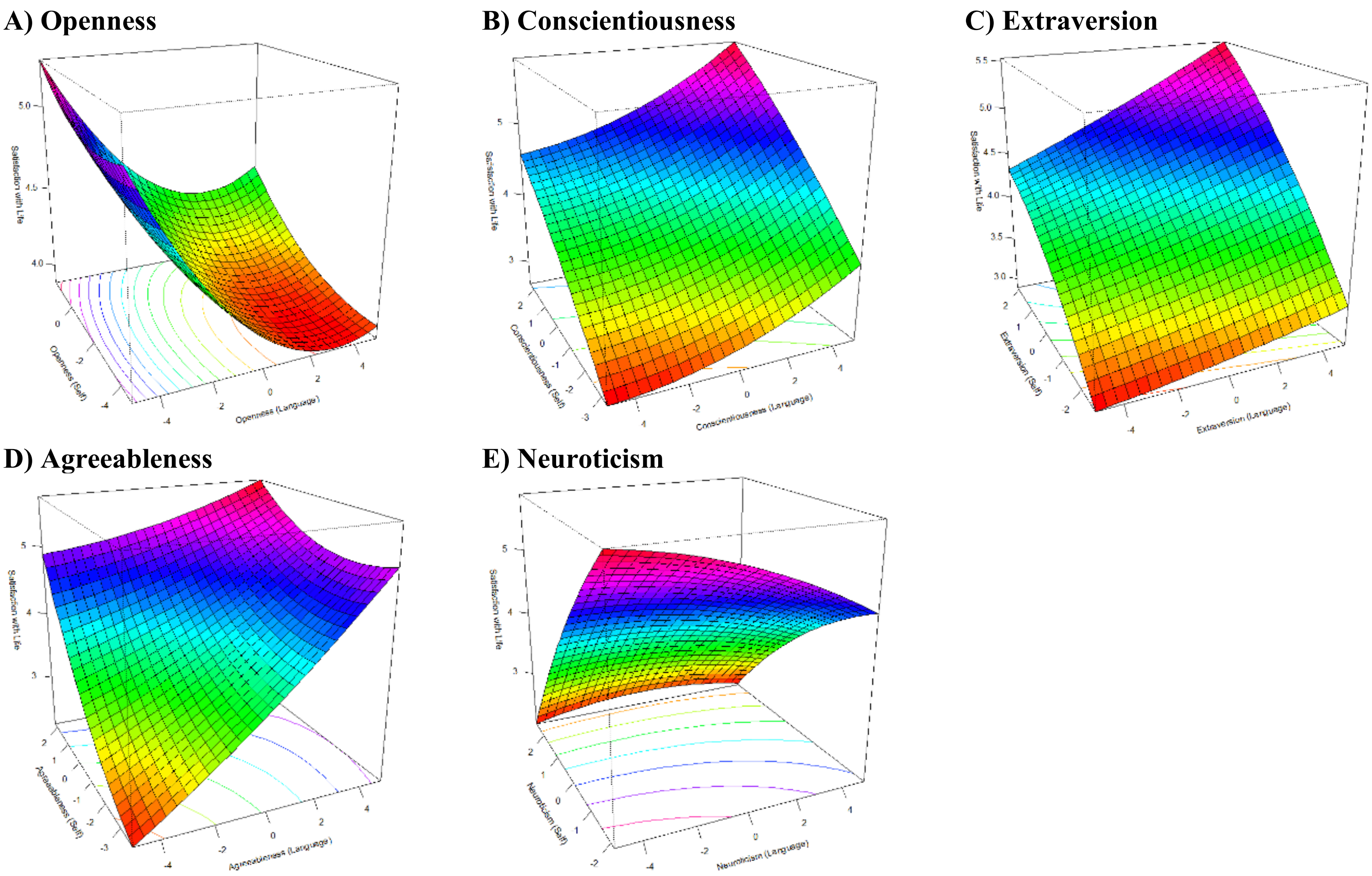

Supplementary Figure 2 Study 1 Response Surface Plots of the Interaction of QA and Personality Traits. Figures represent the response surface plots of the interactions between Quantified Authenticity and Self-reported and Language-predicted Personality Traits on Life Satisfaction. (N = 9,237). Graphs from left-top to bottom-right present the interaction of QA and the following traits: Openness, Conscientiousness, Extraversion, Agreeableness, and Neuroticism (*N* = 3,215).

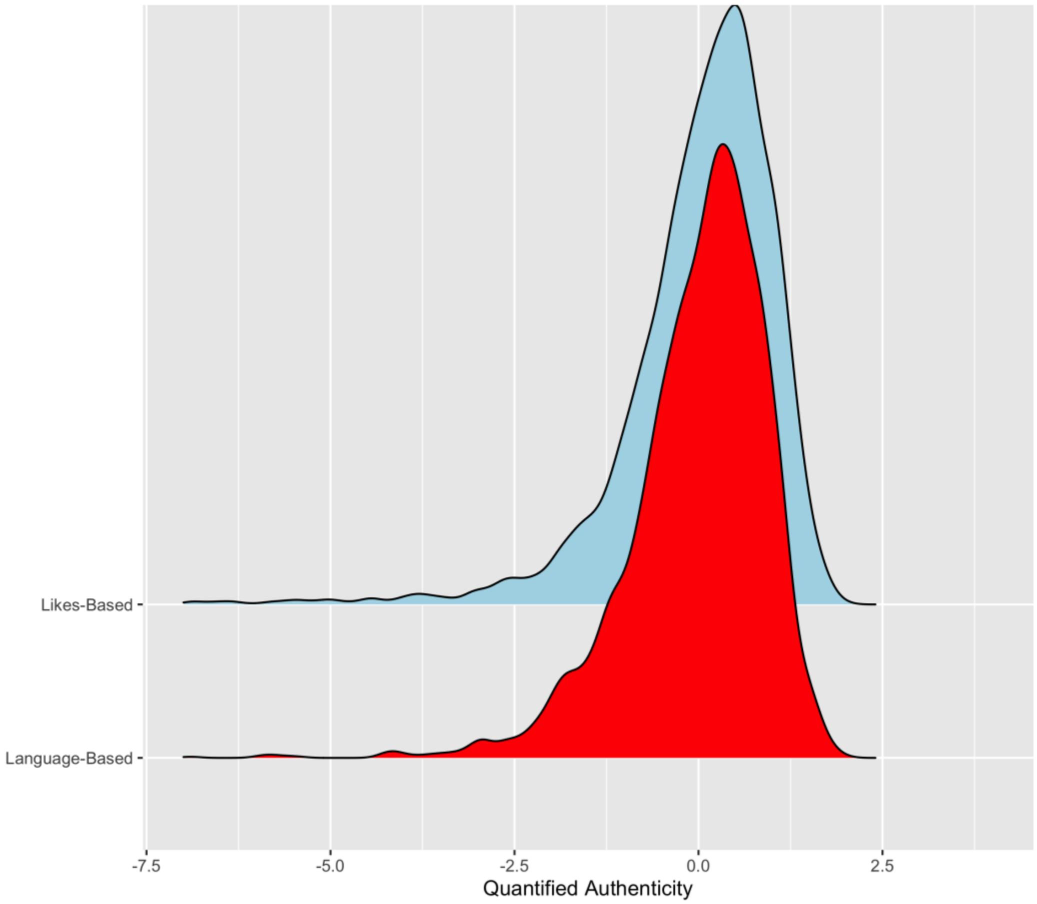

Supplementary Figure 3 Study 1 Standardized Ridgeline Plots of QA. Standardized means are plotted for Quantified Authenticity (QA) scores calculated using Euclidian Distance between self-rated and personality predictions based on Likes and Language. QA scores calculated based on the Likes-model is displayed in the back (indicated in blue); QA scores calculated based on the Language-model is indicated in the front (in red).

A)

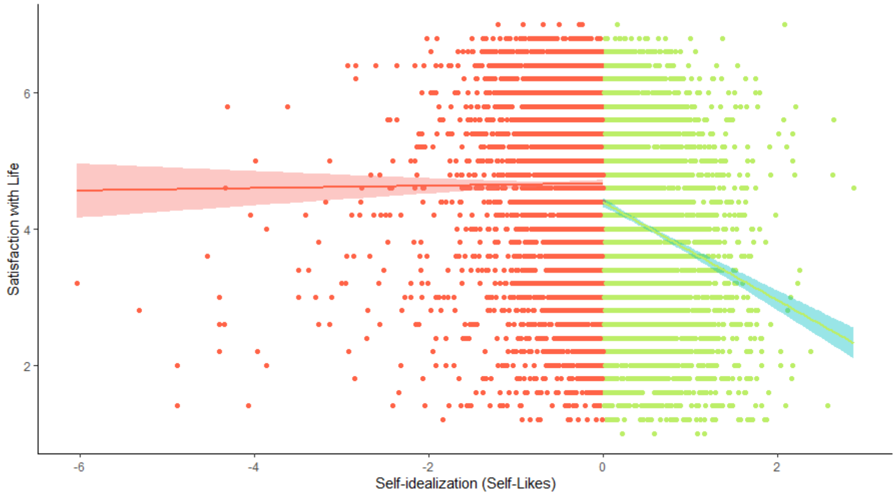

B)

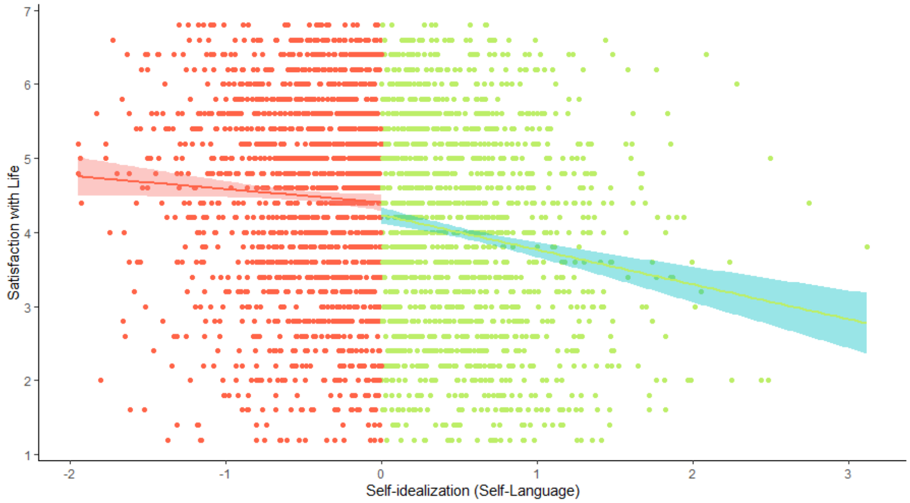

Supplementary Figure 4 Study 1 Relationship between Life Satisfaction and Deviations from Self-Reports. Panels in Figure 4 depict the relationship between Life Satisfaction and normatively self-deprecating deviations from the self-reported profile (red) compared to normatively self-enhancing deviations (green). The top panel is based on the Likes-based model (*N* = 9,237) and the bottom panel is based on the Language-Based model (*N* = 3,215). The error bands represent a 95% confidence interval.

F. Supplementary Tables – Study 1

**Measure**	***M***	***SD***	**1**	**2**	**3**
1. Euclidean Distance (*inverse)*	2.44	1.30			
2. Manhattan Distance (*inverse)*	4.87	3.28	.93		
3. Correlational Similarity	.30	.49	.28	.22	
4. Cosine Similarity	.46	.27	.21	.17	.52

Supplementary Table 1 Study 1 Correlation Table, Likes-Based Model. Supplementary Table 1 presents the Pearson's Correlation of Distance and Similarity Measures, Likes to Self (N = 9,237). All correlations are significant at $p < .001$.

**Measure**	***M***	***SD***	**1**	**2**	**3**
1. Euclidean Distance (*inverse)*	2.52	1.15			
2. Manhattan Distance (*inverse)*	5.06	2.89	.90		
3. Correlational Similarity	.31	.48	.46	.35	
4. Cosine Similarity	.47	.26	.31	.23	.52

Supplementary Table 2 Study 1 Correlation Table, Language-Based Model. Supplementary Table 1 presents the Pearson's Correlation of Distance and Similarity Measures, Language to Self (N = 3,215). All correlations are significant at $p < .001$.

	Model 1			Model 2			Model 3		
	B	SE	β	B	SE	β	B	SE	β
				**Likes-Based Model** (*N* = 6,648)					
QA	**.144*** **	.017	.105	**.065*** **	.016	.047	.042	.027	.030
Gender	.069*	.035	.024	.283***	.031	.100	.284***	.031	.100
Age	.004	.017	.003	-.044**	.015	-.315	-.045**	.015	-.033
Extr.	-	-	-	.028	.016	.020	.013	.016	.010
O	-	-	-	-.030*	.015	-.022	-.013	.016	-.009
C	-	-	-	.169***	.016	.122	.179***	.016	.129
E	-	-	-	.128***	.016	.093	.131***	.016	.095
A	-	-	-	.069***	.016	.050	.066***	.017	.047
N	-	-	-	-.562***	.018	-.406	-.573***	.018	-.415
QA×Gender	-	-	-	-	-	-	.018	.032	.011
QA×Age	-	-	-	-	-	-	-.040*	.017	.026
QA×O	-	-	-	-	-	-	.048**	.015	.037
QA×C	-	-	-	-	-	-	.021	.016	.015
QA×E	-	-	-	-	-	-	.005	.017	.004
QA×A	-	-	-	-	-	-	-.027	.015	-.021
QA×N	-	-	-	-	-	-	-.043*	.019	-.031
Adj-R_2		.01			.27			.28	
				**Language-Based Model** (*N* = 2,943)					
QA	**.075** **	.026	.054	.010	.024	.007	.021	.035	.015
Gender	.121*	.052	.042	.292***	.047	.102	.297***	.047	.104
Age	-.011	.026	-.008	-.072**	.022	-.051	-.079***	.023	-.056
Extr.	-	-	-	-.004	.024	-.003	-.002	.025	-.002
O	-	-	-	-.032	.023	-.022	-.025	.024	-.018
C	-	-	-	.156***	.024	.111	.161***	.025	.115
E	-	-	-	.128***	.025	.091	.129***	.025	.092
A	-	-	-	.112***	.024	.080	.110***	.025	.078
N	-	-	-	-.555***	.027	-.394	-.569***	.028	-.405
QA×Gender	-	-	-	-	-	-	.010	.046	.005
QA×Age	-	-	-	-	-	-	-.022	.022	-.018
QA×O	-	-	-	-	-	-	.007	.022	.006
QA×C	-	-	-	-	-	-	.006	.023	.005
QA×E	-	-	-	-	-	-	.008	.025	.006
QA×A	-	-	-	-	-	-	-.012	.024	-.009
QA×N	-	-	-	-	-	-	-.062	.029	-.048
Adj-R_2		.004			.27			.27	

Supplementary Table 3 Study 1 Regression Analysis of Life Satisfaction on Quantified Authenticity. Models presented include Quantified Authenticity (QA-Euclidean Distance) on Life Satisfaction, with Controls, and Interaction Effects of QA and the Big Five Personality Traits and Demographics (Language-Based Model *N* = 3,215; Likes-Based Model *N* = 2,943). ***p < .001, **p < .01, *p < .05, ·p < .10.

	Likes-Based				Language-Based			
	Model 1		Model 2		Model 1		Model 2	
	B	SE	B	SE	B	SE	B	SE
**Euclidean Distance (inverse)**								
Euclidean Dist.	**.156*** **	.014	**.069*** **	.013	**.119*** **	.025	**.046***	.023
Extreme	-	-	.027*	.013	-	-	.005	.023
O	-	-	-.025*	.013	-	-	-.029	.022
C	-	-	.161***	.013	-	-	.157***	.023
E	-	-	.151***	.014	-	-	.150***	.024
A	-	-	.080***	.014	-	-	.123***	.023
N	-	-	-.511***	.015	-	-	-.503***	.026
**Manhattan Distance (inverse)**								
Manhattan Dist.	**.159*** **	.014	**.080*** **	.013	**.159*** **	.025	**.072** **	.023
Extreme	-	-	.026*	.013	-	-	.009	.023
O	-	-	-.024	.013	-	-	-.028	.022
C	-	-	.161***	.013	-	-	.153***	.023
E	-	-	.149***	.014	-	-	.145***	.024
A	-	-	.082***	.014	-	-	.123***	.023
N	-	-	-.511***	.015	-	-	-.503***	.025
**Cosine Similarity**								
Cosine Sim.	**.056*** **	.014	.007	.013	.003	.025	.003	.022
Extreme	-	-	.004	.013	-	-	-.011	.022
O	-	-	-.026*	.013	-	-	-.029	.022
C	-	-	.165***	.013	-	-	.158***	.023
E	-	-	.151***	.014	-	-	.152***	.024
A	-	-	.082***	.014	-	-	.123***	.023
N	-	-	-.516***	.015	-	-	-.504***	.026
**Correlational Similarity**								
Correlational Sim.	**.127*** **	.014	**.027***	.013	.020	.025	.027	.022
Extreme	-	-	.002	.013	-	-	-.014	.022
O	-	-	-.025	.013	-	-	-.028	.022
C	-	-	.163***	.013	-	-	.158***	.023
E	-	-	.152***	.014	-	-	.152***	.024
A	-	-	.082***	.014	-	-	.122***	.023
N	-	-	-.513***	.015	-	-	-.504***	.026

Supplementary Table 4 Study 1 Regression Analysis of Life Satisfaction on Four-QA Measures. Study 1. Regression Analysis of Life Satisfaction on Four Different Specifications of Quantified Authenticity (QA) for the Likes-Based Model (N = 9,237) and the Language-Based Model (N = 3,215). ***p < .001, **p < .01, *p < .05, ·p < .10.

	Likes-Based				Language-Based			
	Model 1		Model 2		Model 1		Model 2	
	B	SE	B	SE	B	SE	B	E
				**QA-Euclidean Distance (inverse)**				
QA	**.172*****	.019	**.104*****	.017	**.239*****	.034	**.136*****	.031
Extreme	-	-	.024	.031	-	-	.051	.032
O	-	-	-.040	.033	-	-	-.054	.033
C	-	-	.142***	.033	-	-	.144***	.033
E	-	-	.148***	.032	-	-	.153***	.032
A	-	-	.159***	.032	-	-	.158***	.032
N	-	-	-.495***	.035	-	-	-.506***	.035

Supplementary Table 5 Study 1 Regression Analysis of Life Satisfaction on Common Participants. Regression Analysis of Life Satisfaction Predicted by Quantified Authenticity (QA) for Subset of Common Participants in Likes-Based and Language-Based Models (N = 1,711). ***p < .001, **p < .01, *p < .05, ·p < .10.

Variable	1	2	3	4	5	6	7	8	9	10	11
1. QA (La)											
2. QA (Li)	.29**										
3. Openness (La)	-.03	-.11**									
4. Openness (Li)	-.02	.17**	.33**								
5. Conscientiousness (La)	.07**	.18**	-.26**	-.11**							
6. Conscientiousness (Li)	.02	.49**	-.19**	-.05*	.42**						
7. Extraversion (La)	.07**	.14**	-.05*	.01	.34**	.18**					
8. Extraversion (Li)	.08**	.33**	-.15**	-.12**	.27**	.44**	.39**				
9. Agreeableness (La)	.13**	.10**	-.02	-.15**	.31**	.24**	.24**	.18**			
10. Agreeableness (Li)	.09**	.36**	-.05*	-.10**	.25**	.45**	.10**	.32**	.36**		
11. Neuroticism (La)	-.14**	-.18**	.18**	.05*	-.34**	-.24**	-.31**	-.17**	-.37**	-.11**	
12. Neuroticism (Li)	-.09**	-.46**	.13**	.06*	-.25**	-.53**	-.14**	-.41**	-.19**	-.36**	.37**

Supplementary Table 6 Study 1 Correlation Table of Common Participants. The table presents Pearson's correlation between each of the variables in the participants present in both the Language- and Likes-Based Models (N =1,711). "La" refers to a variable calculated using the Language-based model and "Li" refers to a variable calculated using the Likes-based model. * indicates $p < .05$. ** indicates $p < .01$.

G. Supplementary Tables – Study 2

Group	*Initial Pool*	*Completed Survey Responses*			*Excluded*	*Final Sample*
		Survey 1	*Survey 2*	*Survey 3*		
A	100	83	67	62	(21)	41
B	100	84	72	67	(18)	49

Supplementary Table 7 Study 2 Attrition. Supplementary Table 7 presents participants across the duration of Study 2.

	**SWL**	**Positive Affect**	**Negative Affect**	**Mood**
(Intercept)	-.056	-.070	.082	-.024
	(.157)	(.157)	(.157)	(.157)
Auth. Condition	.102	.129	-.150	.044
	(.213)	(.212)	(.212)	(.213)
R_2	.003	.004	.006	.000
Adj. R_2	-.009	-.007	-.006	-.011
Num. obs.	90	90	90	90
RMSE	1.004	1.004	1.003	1.005

Supplementary Table 8 Study 2 Key Outcome Variables By Condition. Ordinary Least Squares regression results using treatment in Week 1 to predict well-being outcomes. "SWL" represents "Satisfaction with Life". "Auth. Condition" is a binary variable where 1 = Authenticity Condition, and 0 = Idealization Condition. $^{***}p < .001$, $^{**}p < .01$, $^{*}p < .05$, $\cdot p < .10$.

	Life Satisfaction			*Positive Affect*			*Negative Affect*			*Mood*		
(Intercept)	-.125	**1.776***	.755	-.275$^{\cdot}$	-.330	**-3.038***	.275$^{\cdot}$	-.832	-1.205	-.245	.746	-.540
	(.156)	(.802)	(1.276)	(.152)	(.819)	(1.175)	(.152)	(.809)	(1.171)	(.153)	(.821)	(1.317)
Auth. Condition	.230	.242	.117	**.505***	**.542***	**.423***	**-.506***	**-.491***	**-.391***	**.451***	**.463***	.378$^{\cdot}$
	(.211)	(.207)	(.207)	(.206)	(.212)	(.190)	(.206)	(.209)	(.190)	(.207)	(.212)	(.213)
Gender		-.173	-.072		-.154	-.185		.273	-.025		-.291	-.261
		(.240)	(.249)		(.245)	(.229)		(.242)	(.228)		(.245)	(.256)
Age		**-.069****	-.047$^{\cdot}$		.014	.041$^{\cdot}$		.027	.004		-.021	-.004
		(.025)	(.026)		(.026)	(.024)		(.026)	(.024)		(.026)	(.027)
Openness			.097			-.010			.053			.006
			(.145)			(.133)			(.133)			(.149)
Conscientiousness			.138			-.018			-.017			-.030
			(.134)			(.123)			(.123)			(.138)
Extraversion			.200			**.548*****			.086			.282$^{\cdot}$
			(.144)			(.132)			(.132)			(.148)
Agreeableness			-.171			.187			-.132			.108
			(.173)			(.159)			(.159)			(.179)
Neuroticism			-.202			-.148			**.508*****			-.167
			(.128)			(.118)			(.117)			(.132)
R^2	.013	.098	.212	.064	.077	.345	.064	.083	.337	.051	.072	.175
Adj. R^2	.002	.066	.132	.053	.044	.279	.053	.050	.270	.040	.039	.092
Num. obs.	90	88	88	90	88	88	90	88	88	90	88	88
RMSE	.999	.965	.931	.973	.986	.857	.973	.974	.854	.980	.988	.960

Supplementary Table 9 Study 2 Week 1 Well-Being Outcomes By Condition. Supplementary Table 9 presents the results of Ordinary Least Squares regression of condition assignment on well-being outcomes in Week 2. “Auth. Condition” is a binary variable where 1 = Authenticity Condition and 0 = Idealization Condition. ***p < .001, **p < .01, *p < .05, ·p < .10.

H. Supplementary References

1. Matz, S. C., Gladstone, J. J. & Stillwell, D. Money Buys Happiness When Spending Fits Our Personality. *Psychol. Sci.* **27**, 715–725 (2016).
2. DeNeve, K. M. & Cooper, H. The happy personality: A meta-analysis of 137 personality traits and subjective well-being. *Psychol. Bull.* **124**, 197 (1998).
3. Schimmack, U., Oishi, S., Furr, R. M. & Funder, D. C. Personality and Life Satisfaction: A Facet-Level Analysis. *Personal. Soc. Psychol. Bull.* **30**, 1062–1075 (2004).
4. Schimmack, U., Radhakrishnan, P., Oishi, S., Dzokoto, V. & Ahadi, S. Culture, personality, and subjective well-being: Integrating process models of life satisfaction. *J. Pers. Soc. Psychol.* **82**, 582 (2002).
5. Simonsohn, U., Simmons, J. P. & Nelson, L. D. Specification Curve: Descriptive and Inferential Statistics on All Reasonable Specifications. *SSRN Electron. J.* (2015). doi:10.2139/ssrn.2694998
6. Youyou, W., Kosinski, M. & Stillwell, D. Computers judge personalities better than humans. *Proc. Natl. Acad. Sci.* **112**, 1036–1040 (2015).
7. Park, G. *et al.* Automatic personality assessment through social media language. *J. Pers. Soc. Psychol.* **108**, 934 (2015).
8. Vazire, S. & Gosling, S. D. e-Perceptions: Personality Impressions Based on Personal Websites. *J. Pers. Soc. Psychol.* **87**, 123–132 (2004).
9. Bailey, E. Authenticity and Well-being | OSF Registries. (2020). Available at: https://osf.io/njcgs.
10. Kavcıoğlu, F. C. The role of meta-mood experience on the mood-congruency effect in recognizing emotions from facial expressions. *Unpubl. Dr. Diss. Middle East Tech. Univ. Sch. Soc. Sci. Ankara.* (2011).
11. Mayer, J. D., Gaschke, Y. N., Braverman, D. L. & Evans, T. W. Mood-congruent judgment is a general effect. *J. Pers. Soc. Psychol.* **63**, 119–132 (1992).
12. DeWall, C. N., Maner, J. K. & Rouby, D. A. Social exclusion and early-stage interpersonal perception: Selective attention to signs of acceptance. *J. Pers. Soc. Psychol.* **96**, 729 (2009).
13. Ramsburg, J. T. & Youmans, R. J. Meditation in the higher-education classroom: Meditation training improves student knowledge retention during lectures. *Mindfulness (N. Y).* **5**, 431–441 (2014).
14. Hershenberg, R., Davila, J. & Leong, S. H. Depressive symptoms in women and the preference and emotional benefits of discussing positive life events. *J. Soc. Clin. Psychol.* **33**, 767–788 (2014).
