# Supplementary material for: Authentic self-expression on social media is associated with greater subjective well-being
Source: Nat Commun. 2020 Oct 6;11:4889. doi: 10.1038/s41467-020-18539-w (PMC7538578; doi:10.1038/s41467-020-18539-w)
Supplement: Supplementary file 3 — Reporting Summary [file 41467_2020_18539_MOESM3_ESM.pdf]

nature research

Corresponding author(s): Erica Bailey, Sandra Matz

Last updated by author(s): Jun 25, 2020

Reporting Summary

Nature Research wishes to improve the reproducibility of the work that we publish. This form provides structure for consistency and transparency in reporting. For further information on Nature Research policies, see our Editorial Policies and the Editorial Policy Checklist.

Statistics

For all statistical analyses, confirm that the following items are present in the figure legend, table legend, main text, or Methods section.

n/a	Confirmed	
☐	☒	The exact sample size (n) for each experimental group/condition, given as a discrete number and unit of measurement
☐	☒	A statement on whether measurements were taken from distinct samples or whether the same sample was measured repeatedly
☐	☒	The statistical test(s) used AND whether they are one- or two-sided<br>*Only common tests should be described solely by name; describe more complex techniques in the Methods section.*
☐	☒	A description of all covariates tested
☐	☒	A description of any assumptions or corrections, such as tests of normality and adjustment for multiple comparisons
☐	☒	A full description of the statistical parameters including central tendency (e.g. means) or other basic estimates (e.g. regression coefficient) AND variation (e.g. standard deviation) or associated estimates of uncertainty (e.g. confidence intervals)
☐	☒	For null hypothesis testing, the test statistic (e.g. F, t, r) with confidence intervals, effect sizes, degrees of freedom and P value noted<br>*Give P values as exact values whenever suitable.*
☒	☐	For Bayesian analysis, information on the choice of priors and Markov chain Monte Carlo settings
☒	☐	For hierarchical and complex designs, identification of the appropriate level for tests and full reporting of outcomes
☐	☒	Estimates of effect sizes (e.g. Cohen's d, Pearson's r), indicating how they were calculated

Our web collection on statistics for biologists contains articles on many of the points above.

Software and code

Policy information about availability of computer code

Data collection	Data for Study 1 was collected using the MyPersonality website application (Stillwell & Kosinski, 2004). Data for Study 2 was collected through the electronic survey website Qualtrics.
Data analysis	Data was analyzed using R (Version 1.2.1335)

For manuscripts utilizing custom algorithms or software that are central to the research but not yet described in published literature, software must be made available to editors and reviewers. We strongly encourage code deposition in a community repository (e.g. GitHub). See the Nature Research guidelines for submitting code & software for further information.

Data

Policy information about availability of data

All manuscripts must include a data availability statement. This statement should provide the following information, where applicable:

- Accession codes, unique identifiers, or web links for publicly available datasets
- A list of figures that have associated raw data
- A description of any restrictions on data availability

Data is available upon request to the authors.

Field-specific reporting

Behavioural & social sciences study design

All studies must disclose on these points even when the disclosure is negative.

Study description	Study 1 was an archival quantitative cross-sectional study designed to quantify authentic self-expression on social media and estimate it's correlation with subjective well-being. Study 2 was a quantitative experiment designed to examine if the relationship between authenticity and subjective well-being observed in Study 1 was causal such that authenticity increases subjective well-being.
Research sample	The sample for Study 1 was a non-representative sample of Facebook users who used the MyPersonality application and agreed to submit their Facebook data for academic research. We were able to ascertain gender and age for a subset of participants; this included 6,648 participants in the Likes-based model (61.24% female, mean age = 24.97 years, SD age = 8.42 years) and 2,943 participants in the Language-based model (58.27% female, mean age = 25.97 years, SD age = 10.42 years). Sample size was determined based on data availability.<br><br>Study 2 was a non-representative sample of college students who indicated they were active social media users (mean age = 22.98 years old, SD age = 4.17 years, 73.86% female). We aimed to recruit 200 students based on financial constraints, however data collection was halted at the onset of the COVID-19 pandemic and the closure of the campus research lab.
Sampling strategy	Study 1 was an archival, cross-sectional, survey-based study limited based on data availability. Study 2 was an experiment, where participants were randomly assigned to one of two conditions. The sample size for Study 2 was originally determined based on financial limitations; data collection was halted at the onset of the COVID-19 pandemic and the closure of the campus research lab.
Data collection	In both studies, data were collected at a time and place of the participants choosing on an electronic device. In Study 1, data was collected using the MyPersonality application. The original data collection was not related to the present research question. In Study 2, data was collected using the online platform Qualtrics. The researcher was not blind to the condition or hypotheses.
Timing	Data for Study 1 were collected between 2007-2012.<br><br>Data for Study 2 were collected in February 2020.
Data exclusions	39 participants were excluded from Study 2. As defined in our per-registration plan, we excluded participants who indicated at the end of the two weeks that they did not take the study seriously.
Non-participation	In Study 2, 71 participants dropped out (did not complete all check-in surveys).
Randomization	Study 1 did not involve randomization. Therefore to estimate the robustness of our findings, we included a number of control variables including the participant's self-reported personality traits and their most extreme trait. In a subsample, presented in the Supplementary Information, we were able to obtain gender and age which were used as additional controls. Our findings were largely robust to these control variables. In Study 2, participants were randomly assigned to one of two conditions.

Reporting for specific materials, systems and methods

We require information from authors about some types of materials, experimental systems and methods used in many studies. Here, indicate whether each material, system or method listed is relevant to your study. If you are not sure if a list item applies to your research, read the appropriate section before selecting a response.

Materials & experimental systems

n/a	Involved in the study	
x		Antibodies
x		Eukaryotic cell lines
x		Palaeontology and archaeology
x		Animals and other organisms
	x	Human research participants
x		Clinical data
x		Dual use research of concern

Methods

n/a	Involved in the study	
x		ChIP-seq
x		Flow cytometry
x		MRI-based neuroimaging

Human research participants

Policy information about studies involving human research participants

Population characteristics	See above
Recruitment	In Study 1, participants were users of a Facebook application which involved personality assessments, therefore participants self-selected into the study. The purpose of the app was to give participants information about their personality, thus incentivizing honest responses. Additionally, participant personality expression was obtained from their Facebook profiles, providing ecological validity. This data set is limited to social media users and therefore cannot be generalized to those who do not use social media.<br><br>In Study 2, participants were recruited through a University subject pool, and pre-screened for social media use. They self-selected into a study about social media, however were not told the explicit hypotheses of the study. This self-selection and sourcing of participants means that the observed sample may be younger and have a greater interest in social media related research than the average population. It may be that the effects of the treatments are overstated in the younger sample size. However, they may also be understated, given the importance and usage rates of social media among younger individuals.
Ethics oversight	Both studies were reviewed by the Columbia University Human Research Protection Office and Institutional Review Board (IRB). They determined that Study 1 did not qualify as Human Subjects Research because the data was archival. The procedures of Study 2 were approved by Columbia's IRB.

Note that full information on the approval of the study protocol must also be provided in the manuscript.
